# An automatic method for counting wheat tiller number in the field with terrestrial LiDAR

**DOI:** 10.1186/s13007-020-00672-8

**Published:** 2020-09-29

**Authors:** Yuan Fang, Xiaolei Qiu, Tai Guo, Yongqing Wang, Tao Cheng, Yan Zhu, Qi Chen, Weixing Cao, Xia Yao, Qingsong Niu, Yongqiang Hu, Lijuan Gui

**Affiliations:** 1 National Engineering and Technology Center for Information Agriculture, Key Laboratory for Crop System Analysis and Decision Making, Ministry of Agriculture, Jiangsu Key Laboratory for Information Agriculture, Jiangsu Collaborative InnovationCenter for Modern Crop Production, Nanjing Agricultural University, Nanjing, China; 2 Department of Geography and Environment, University of Hawai`I At Mānoa, 422 Saunders Hall, 2424 Maile Way, Honolulu, HI 96822 USA; 3 Qinghai Science and Technology Information Research Institute Co.Ltd, Xining, Qinghai China

**Keywords:** Terrestrial laser scanning (TLS), Tiller number, Adaptive layering (AL) algorithm, Hierarchical clustering (HC) algorithm, Automatic method, Wheat

## Abstract

**Background:**

The tiller number per unit area is one of the main agronomic components in determining yield. A real-time assessment of this trait could contribute to monitoring the growth of wheat populations or as a primary phenotyping indicator for the screening of cultivars for crop breeding. However, determining tiller number has been conventionally dependent on tedious and labor-intensive manual counting. In this study, an automatic tiller-counting algorithm was developed to estimate the tiller density under field conditions based on terrestrial laser scanning (TLS) data. The novel algorithm, which is named ALHC, involves two steps: (1) the use of an adaptive layering (AL) algorithm for cluster segmentation and (2) the use of a hierarchical clustering (HC) algorithm for tiller detection among the clusters. Three field trials during the 2016–2018 wheat seasons were conducted to validate the algorithm with twenty different wheat cultivars, three nitrogen levels, and two planting densities at two ecological sites (Rugao & Xuzhou) in Jiangsu Province, China.

**Result:**

The results demonstrated that the algorithm was promising across different cultivars, years, growth stages, planting densities, and ecological sites. The tests from Rugao and Xuzhou in 2016–2017 and Rugao in 2017–2018 showed that the algorithm estimated the tiller number of the wheat with regression coefficient (R^2^) values of 0.61, 0.56 and 0.65, respectively. In short, tiller counting with the ALHC generally underestimated the tiller number and performed better for the data with lower plant densities, compact plant types and the jointing stage, which were associated with overlap and noise between plants and inside the dense canopy.

**Conclusions:**

Differing from the previous methods, the ALHC proposed in this paper made full use of 3D crop information and developed an automatic tiller counting method that is suitable for the field environment.

## Background

Wheat is one of the primary food crops, feeding more than half of the world’s population [1, 2]. With a predicted world population of 9 billion in 2050, the demand for wheat is expected to increase by 60%–110% [3–5]. To meet this demand, the annual wheat yield increment must rise from the current level of below 1% to at least 1.6% [4, 5]. At present, wheat yield mainly relies on the increase in grain yield per unit area [6]. The increase in productive tiller number generally enhances yield potential over a range of environmental conditions [7]. Therefore, an accurate and efficient method for acquiring the tiller number in real time under field conditions can be helpful for determining a reasonable group density; assessing the efficiency of crop management, e.g., irrigation and applying fertilizer; or functioning as a phenotyping trait for breeding practices in the field.

To date, the most common method used for measuring tiller numbers is manual counting, which is extremely time-consuming and labor-intensive and is constrained by human error [8]. Remote sensing provides an alternative for the high-throughput evaluation of tiller number and has been widely used in the past several decades [10–16, 29]. The common practice is to establish a linear relationship between the tiller number and spectral features from optical remote sensing imagery. Flowers et al. [10] used spectral information from aerial photographs to predict wheat tiller density through correlation with a series of vegetation indexes (VIs). The results demonstrated that the VIs were reliable indicators for estimating wheat tiller density in the field. Since then, studies have tried to further prove the accuracy and reliability of VIs, especially the normalized difference vegetation index (NDVI), to forecast the tiller number [8–11]. However, the method for measuring tiller number using correlation with VIs is influenced by environmental factors and changes in the external conditions, such as crop varieties and field management.

With the development of image processing technology, image-based methods have been successfully applied to overcome some shortcomings of VIs [10, 11]. Boyle et al. [12] collected RGB images of a single spring wheat plant at different filming angles (0°, 45°, 90°) and calculated the number of wheat tillers using the Frangi filter algorithm. More recently, Constantino et al. [13] used an image processing system, Seight, to measure the height and number of tillers of a single plant, in which the Canny edge detection algorithm was utilized to detect the tillers. However, similar to the VIs, the counting accuracy with images from two-dimensional (2D) cameras is constrained by the illumination conditions at the time of image acquisition. Moreover, RGB images lack spatial and volumetric information, which is more closely related to plant function and yield-related traits [14].

More advanced than 2D RGB cameras, some sensors have the capability to collect three-dimensional information from the target objects. Innovative research was performed by Yang et al. [15] in which a high-throughput system (H-SMART) based on a conventional X-ray computed tomography (CT) system that was developed to automatically measure rice tillers could achieve a performance with a mean absolute error (MAE) of 0.22 at the tillering stage. Despite the high accuracy, the CT system requires a rigorous experimental environment to ensure the safety of the experimenter and to prevent genetic mutation in experimental objects. Inspired by the work of Yang’s team, Huang et al. [16] applied magnetic resonance imaging (MRI), which features nondestructive methods without radiation to acquire the number of rice tillers. However, these experiments are only applicable to the laboratory or the assembly line, and very few studies have explored methods to acquire tiller information automatically and efficiently in field environments.

Light detection and ranging (LiDAR), which is an active sensor, overcomes many of the disadvantages of passive sensing because it is capable of operation regardless of the ambient light conditions and directly provides highly accurate three-dimensional (3D) information [17–19]. While LiDAR use in forestry has been well established for decades [20–23], its application to cereal crops is still in the early stages [24]. Recently, LiDAR has drawn extensive attention in plant phenotyping, which has made the method popular as the key technical component for developing next-generation plant phenotyping techniques [19]. Currently, research is focused on rapid determination of biomass, plant height, and leaf area [20, 24–28]. However, there are few reports about the detection of tiller number using LiDAR. To our knowledge, Guo et al. [29] developed a high-throughput crop phenotyping platform, Crop 3D, which included a fractional module that used LiDAR to scan single rice plants into point clouds. The classical k-means clustering algorithm was used to calculate the tiller number, with an accuracy (R^2^) that reached 0.80. Although the k-means algorithm is simple and efficient, it is necessary to define the number of classes in advance, which does not apply to cases where the class number is unknown. Moreover, unlike the individual plants on the assembly line in Guo’s research, the canopy is much denser in the field, which is compounded by other uncontrollable factors such as wind, which also means much more severe occlusion between plants. Overall, direct tiller segmentation or detection in a field population based on large quantities of LiDAR point data and the inevitable accompanying noise is still a major challenge.

Therefore, the main objectives of this study were (1) to propose an automatic approach suitable for counting tiller number under field conditions based on TLS data for wheat and (2) to validate the applicability of the method under different treatments and growth stages. For the second objective, different wheat cultivars, plant densities, and nitrogen fertilization rates were designed in three field trials. This method is expected to provide a new perspective for the detection of wheat tiller number in three dimension and to enhance the practical use of TLS in precision agriculture.

## Methods

### Plant materials and growth conditions

#### 2016–2017 field trial configuration

Experiment A (Exp. A): The field trial was conducted at the Rugao Experimental Demonstration Base (32°15′ N, 120°38′ E) of the National Information Agricultural Engineering Technology Center in Rugao City, Jiangsu Province. Two wheat cultivars, ‘Yangmai 15′ (V1) and ‘Yangmai 16′ (V2), were selected to represent compact and diffuse plant types, respectively. Three nitrogen rates of 0 kg/ha (N0), 150 kg/ha (N1, 484.91 g/plot), and 300 kg/ha (N2, 969.83 g/plot) were set, among which N1 was consistent with the average nitrogen level. Fifty percent of the N fertilizers were applied on the sowing day, and 50% were applied at the jointing stage. Two planting density levels were set for the experiment: 25 cm (2.4 × 10^6^ seedlings/ha) for D1 and 40 cm (1.5 × 10^6^ seedlings/ha) for D2, which corresponded to 26 rows/plot and 17 rows/plot. For each set of growing conditions, the experimental design was established with randomized blocks with three replicates, for a total of 36 plots, each of which had an area of 30 m^2^ (6 m × 5 m).

Experiment B (Exp. B): The second field trial was conducted at the Xuzhou Agricultural Research Institute in Xuzhou City, Jiangsu Province (34°19′ N, 117°2′ E). A total of 17 varieties were used: ‘Jimai 211′, ‘Saidema 5′, ‘Cunmai 11′, ‘Zhengmai 119′, ‘Loumai 956′, ‘Anke 1405′, ‘Zhumai 328′, ‘Xinong 528′, ‘Ruihua 1426′, ‘Luomai 6010′, ‘Xinong 501′, ‘Huaichuan 365′, ‘Zhengmai 1836′, ‘Zhumai 305′, ‘Xinong 364′, ‘Zhoumai 18CK’, and ‘Yanzhan 4110CK1′. In this trial, the nitrogen rate was set at 225 kg/ha (348.60 g/plot). Fifty percent of the N fertilizers were applied on the sowing day, and 50% were applied at the jointing stage. The field density was set with a unified line spacing (23 cm, 2.2 × 10^6^ seedlings/ha, 38 rows/plot). The experimental design was established in randomized blocks with three replicates for a total of 51 plots, each of which had an area of 13 m^2^ (8.3 m × 1.6 m).

### 2017–2018 field trial configuration

Experiment C (Exp. C): The field trial was conducted at the Rugao Experimental Demonstration Base. Two wheat cultivars, ‘Shengxuan 6′ (V1) and ‘Yangmai 16′ (V2), were selected to represent compact and diffuse plant types, respectively. All other experimental conditions were identical to those in Exp. A.

**Fig. 1:**
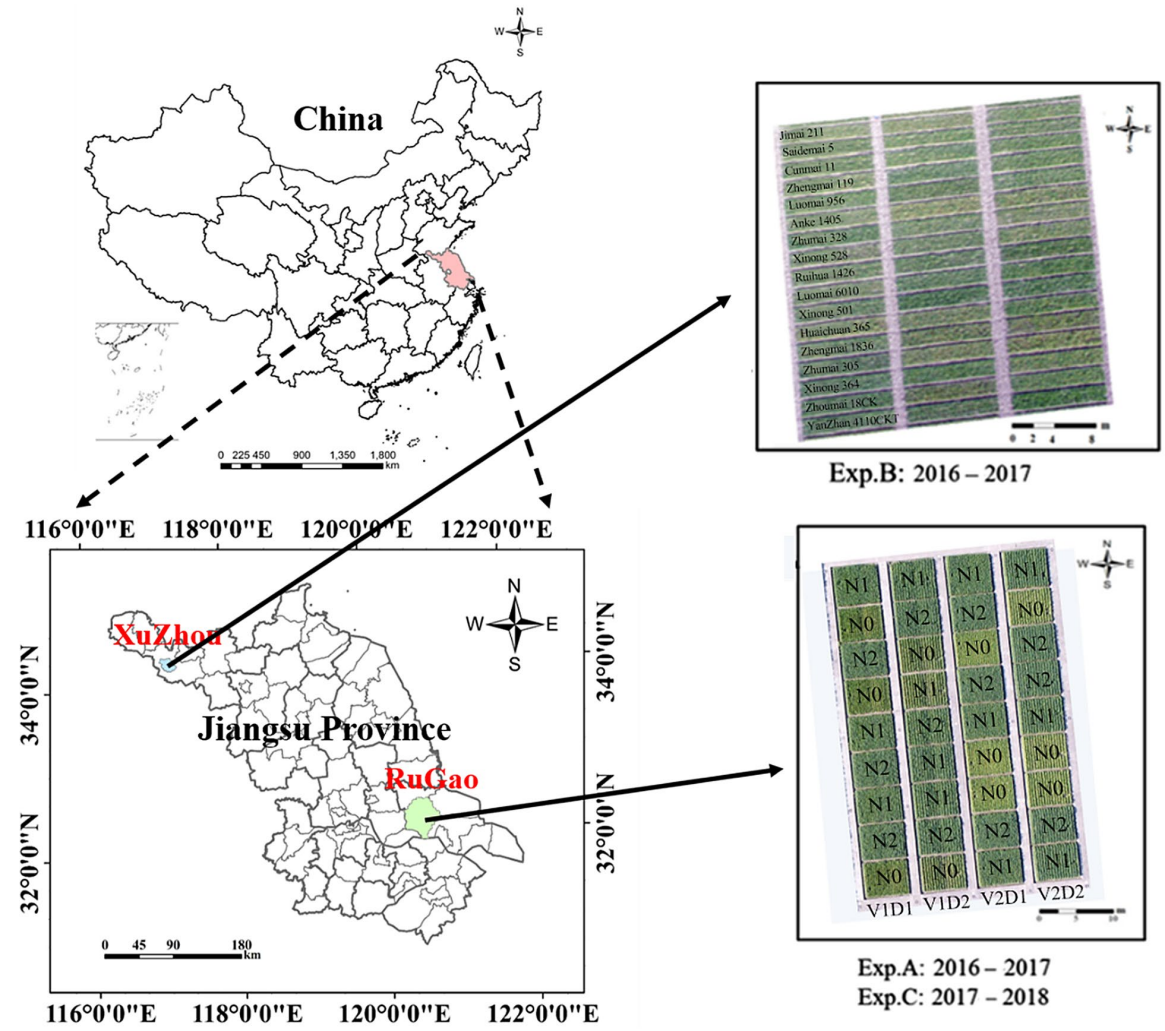
Images of the field experiment area in Rugao (Exp. A &C with two varieties, two planting density levels and three nitrogen rates) & Xuzhou (Exp. B with the same nitrogen rate and planting density level for 17 varieties); the left: the location of experiment sites; the right: the orthophotos captured by the UAV system

**Fig. 2:**
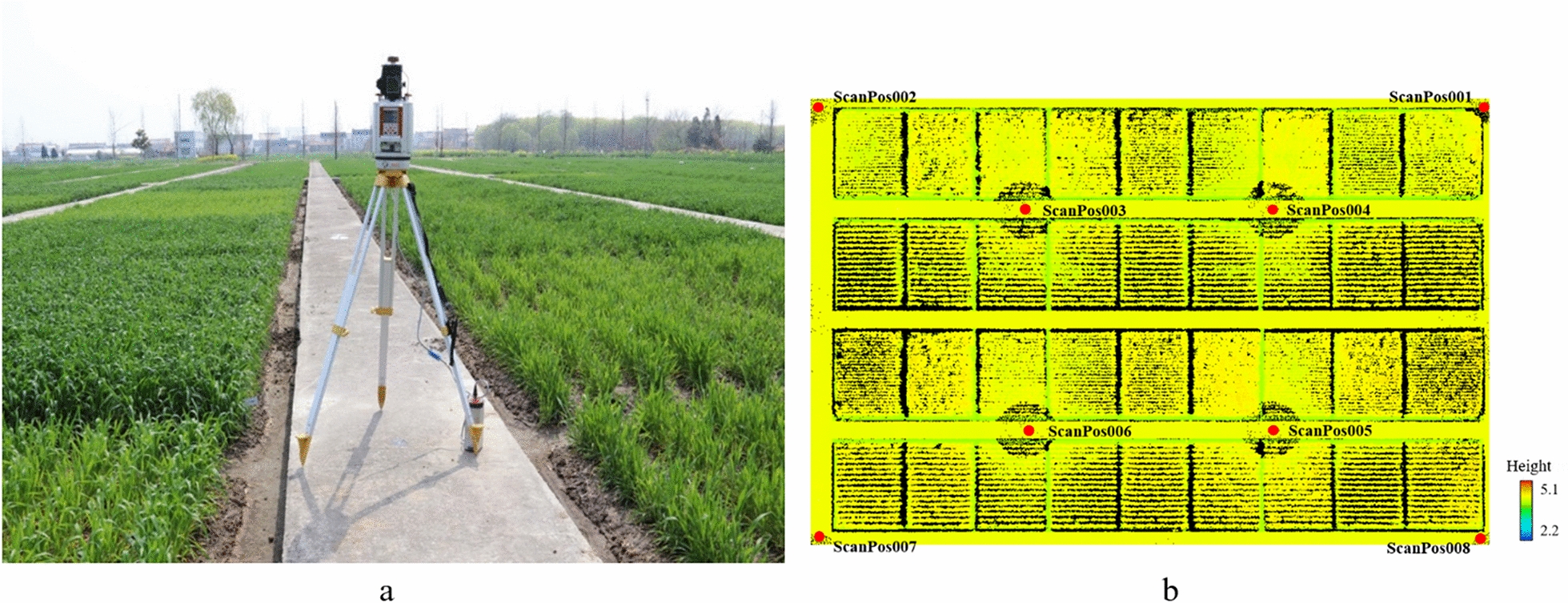
**a** RIEGL-VZ 1000 working in the field at Rugao at a height of 1.5 m; **b** the TLS data of the whole experimental area (the jointing stage, Rugao, 2017–2018) displayed in the supporting software RiCSAN PRO; ScanPos001-ScanPos008 represent the locations of the sites (colors indicate the canopy height); a circular blind zone with a diameter of 1 m around the sites

The specific conditions for each trial (Exp. A, B, C) are shown in Table 1. Orthophotographs of the trials (A, B, C) are shown in Figs. 1, 2.

**Table 1:** Details of the field experiments

Number	Ecological Site	Year	Number of Varieties	Nitrogen Rate (kg/ha)	Planting Density (cm & rows/plot)	Area of plot (m^2^)	Number of plots
Exp. A	Rugao	2016–2017	2	0/150/300	25 (26)	30	36
					40 (17)		
Exp. B	Xuzhou	2016–2017	17	225	23 (38)	13.3	51
Exp. C	Rugao	2017–2018	2	0/150/300	25 (26)	30	36
					40 (17)

### Data acquisition

#### Terrestrial laser scanning measurements

The terrestrial LiDAR system used in this study is a RIEGL-VZ 1000 (RIEGL, Austria, https://www.riegl.com), which is a pulsed 3D scanner that emits near-infrared lasers. The instrument operates on the principle that laser pulses are emitted from the instrument and, using a series of built-in rotating prisms, the laser lights are projected at different angles. The detector receives the signal reflected from the target object and records the target information. The specifications of the RIEGL-VZ 1000 are shown in Table 2.

**Table 2:** The specifications of the RIEGL-VZ 1000

Parameters		Characteristics
Scan performance		
Scan Principle		Pulse type
Laser Wavelength		1550 nm (near infrared)
Angle Resolution		better than 0.0005°
Beam Divergence		0.12 mrad
Scan Precision		5 mm @ 100 m
Pulse Repetition Frequency		3 × 10^5^ pulse/sec
Scan Angle Range		360° × 100° (horizontal × vertical)
Max Measurement Range		1400 m
Min Measurement Range		1 m
General technical data		
Weight		9.8 kg
External Camera		NIKON D810

**Fig. 3:**
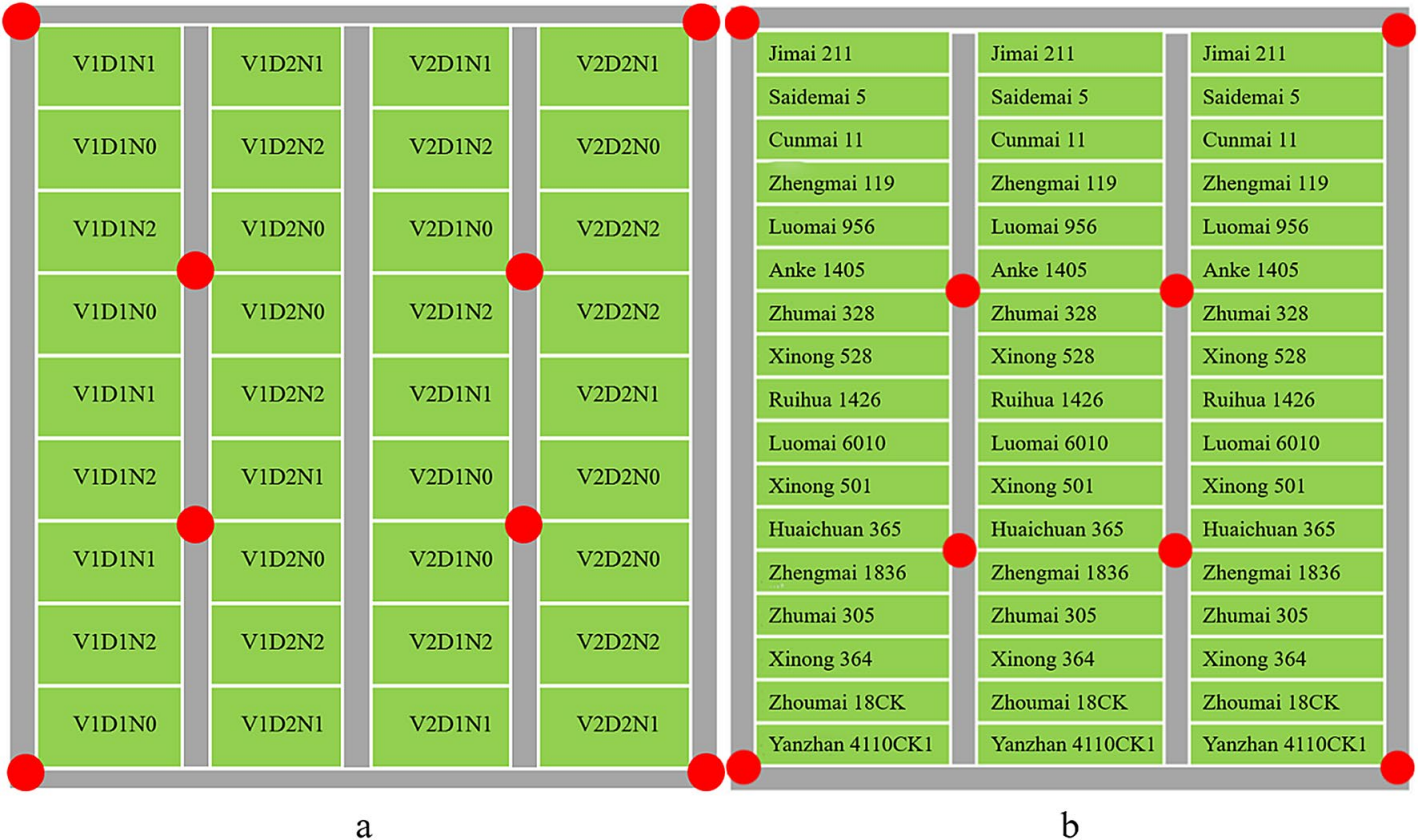
The scanner position schemes: **a** in Rugao and **b** in Xuzhou. The varieties in the set of three rows are consistent; the red solid circle points stand for the scanning positions of the LiDAR instrument. Note: D represents the line spacings (D1: 25 cm, 2.4 × 10^6^ seedlings/ha and D2: 40 cm, 1.5 × 10^6^ seedlings/ha), V represents the varieties (V1: Shengxuan 6 and V2: Yangmai 16), and N represents the three levels of pure nitrogen (N0: 0 kg/ha, N1: 150 kg/ha, N2: 300 kg/ha)

To mitigate the occlusion effects, a multiple-scan scheme was conducted in all trials. According to previous research on the impact of the number and position of the scanning site [30], we settled on an 8-station scanning strategy (Fig. 3). Scanning sites outside the plots could not only supplement the point cloud information of the edge plots, but also obtained sufficient information about surrounding objects. This off-plot information served the registration of the point cloud between different scanning sites. The scan mode was set to mode 60, which indicated an angular resolution of 0.06° and corresponded to a point spacing of 10 cm at the 100 m scanning range.

### Manual field measurements

In all three trials, the total tiller numbers were counted in two one-meter row sections per plot, and then, the number of tillers per unit area was calculated. The manual-counting data were used as the observed value to evaluate the algorithm. The manual measurements were performed at two stages (tillering and jointing), and the terrestrial LiDAR measurements were collected simultaneously (Table 3).

**Table 3:** Summary of the field sampling for the wheat experiments

Experiment	Data type	Method	Growth stage	Number of samples
A	Field measurements	Manual counting	Tillering	36
	TLS data	LiDAR	Jointing	36
B	Field measurements	Manual counting	Tillering	17
	TLS data	LiDAR		
C	Field measurements	Manual counting	Tillering	36
	TLS data	LiDAR	Jointing	36

### Data processing and analysis

The preprocessing of TLS data was carried out in professional software bundled with the scanner. The coordinate registration was the first step. An iterative closest point (ICP) algorithm was applied to register each independent scanner coordinate to the same reference coordinate system. ICP calculated the transformation matrix by a least-squares method based on the corresponding points [31]. The coordinate registration was completed with an average error of 0.006 m for each campaign. Abnormal points floating above, which was caused by insects or small airborne particles, was removed manually. Then we manually intercepted a random row of data from each plot. Finally, the data of each area was exported into separate files.

In this study, the 3D point cloud data for the tillering and jointing stages obtained from the LiDAR measurements are used to estimate the tiller number after preprocessing, e.g., point cloud registration and detour. The processed data for a random row per plot are input into our algorithm to calculate the tiller number of this row (T_row_). Then, the tiller density for the whole plot (T_plot_) is calculated as follows: T_plot_ = T_row_ * r / S where r and S are the number of rows and area of the plot, respectively.

### The automatic tiller-counting algorithm (ALHC)

**Fig. 4:**
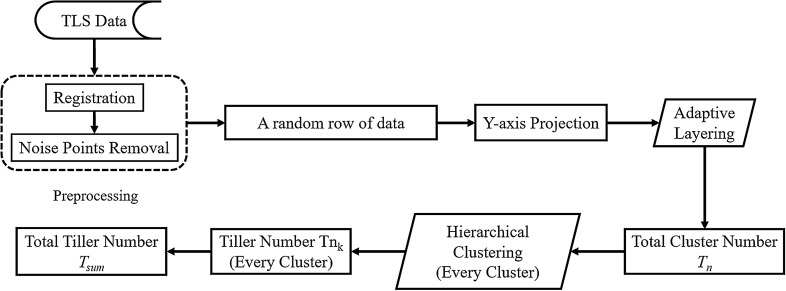
Workflow of the proposed tiller number estimation method (ALHC) using the TLS data

The tiller-counting algorithm employs algorithms in two steps: (1) the adaptive layering (AL) algorithm and (2) the hierarchical clustering (HC) algorithm. We developed our algorithm in MATLAB (version 2016, MathWorks®, USA). A flowchart describing the process is provided in Fig. 4.

### Description of the adaptive layering (AL) algorithm

This step takes a random row of preprocessed point cloud data from each plot as the input, and the number of wheat clusters is the output. In this study, the principle of the adaptive layering algorithm is to identify the interspaces between the wheat stems and to obtain the number of wheat clusters accordingly. A cluster of wheat refers to densely aggregated wheat plants, which usually contain several tillers and stems that may be from the same plant or different plants. It should be noted that the term "stem" here is borrowed for the tillering stage and redefined as the trunk of the aboveground tillers, excluding the leaves.

The specific implementation steps are as follows.

After preprocessing, a random row of wheat point data is obtained for each plot. The X axis is defined as parallel to the direction of the row, which means that the Y axis is perpendicular to the direction of the row and the direction of the plant height is the Z axis.

1. Layering & labeling. A row of wheat points is divided into 1 to n layers according to the plant height (H) from the top to bottom. The first layer (the 1st layer) along the Z axis and the last layer (the n_th_ layer) are removed. The variable ‘n’ is determined as an optimal value based on the degree of overcounting and undercounting of the clusters observed in the layers compared with the planting density after the trial runs. Then, starting from the second layer, the smaller serial number layer is labeled as the ‘leaf’, and the adjacent layer in the sequence is labeled as the ‘stem’. Here, the labels are only used to distinguish among the layers. For example, layers 2 and 3 demonstrate one leaf-stem combination, with 2 as the leaf and 3 as the stem in Fig. 5A b.

**Fig. 5:**
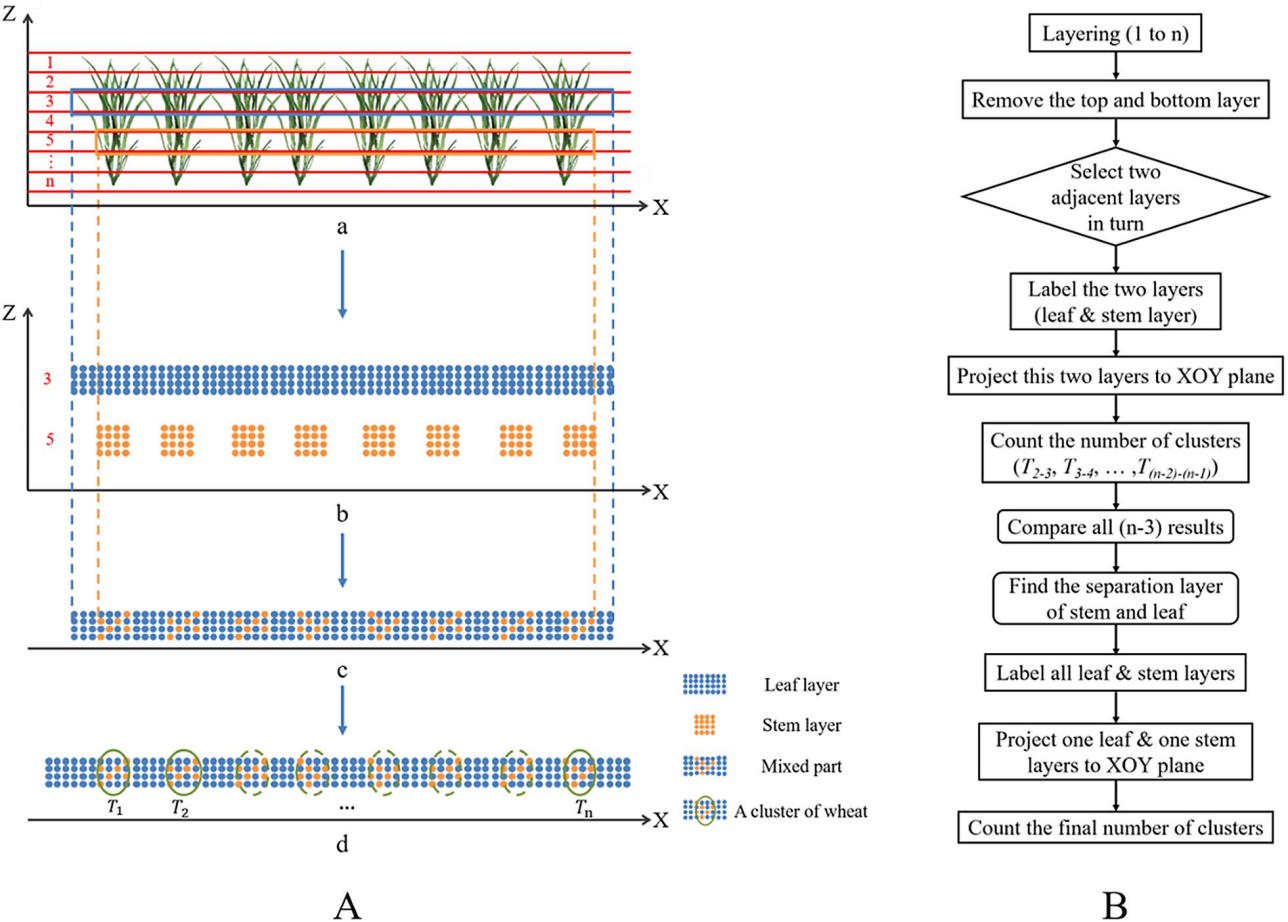
A illustrates the algorithm: **a** one row of wheat was layered; **b** a leaf layer was extracted and then a stem layer was extracted; **c** two layers were superimposed onto one mixed layer; (d) the number of wheat clusters (${T}_{i}$ represented the number of wheat clusters) was calculated. **b** The workflow of the adaptive layering algorithm
2. Iterating. One leaf-stem combination participates in each iteration. The combination is projected along the Z axis to the XOY plane to form one layer of the superimposed point cloud, which retains only the X and Y coordinates of the points. The overlapping leaves allow the leaf point cloud to be considered continuous, while the stem point cloud is discontinuous due to the intervals between the wheat clusters. When the leaf and stem layers are projected along the Z axis to the XOY plane, the intervals are filled by the leaf points called ‘the continuous leaf part’, while the part where the leaf layer meets the stem layer is called ‘the mixed part’ (Fig. 5A c). The criterion for determining a cluster of wheat is to find a continuous leaf part that is located between two adjacent mixed parts. Once an eligible part is detected, the number of clusters (*T*_*i-(i-1)*_, where i represents the layer number) is added. The entire row is traversed to obtain *T*_*i-(i-1)*_, which completes an iteration.
3. Counting. A total of (n-3) iteration results (*T*_*2-3*_, *T*_*3-4*_, *T*_*4-5*_, …, *T*_*(n-2)-(n-1)*_) are compared to determine the leaf layer combination with the largest *T*_*i-(i-1)*_ as the separation layer between the stem and leaf. All layers above the separation layer are marked (excluding itself) as ‘leaf’, and the other layers are marked as ‘stem’. The final output of this step is the result of the iteration in step (2), in which one of the leaf layers and one of the stem layers are selected as the input.
  Thus far, the approximate number of clusters in a row has been calculated.

### Description of the hierarchical clustering (HC) algorithm

An approximate wheat cluster number is obtained in the previous step. When the tillers are too close to each other, they cannot be distinguished with the layering algorithm. Therefore, the hierarchical clustering algorithm is introduced to further calculate the tiller number in each cluster, which may contain several tillers the same plant or different plants.

The specific steps used in the hierarchical clustering [32–34] are as follows.

**Fig. 6:**
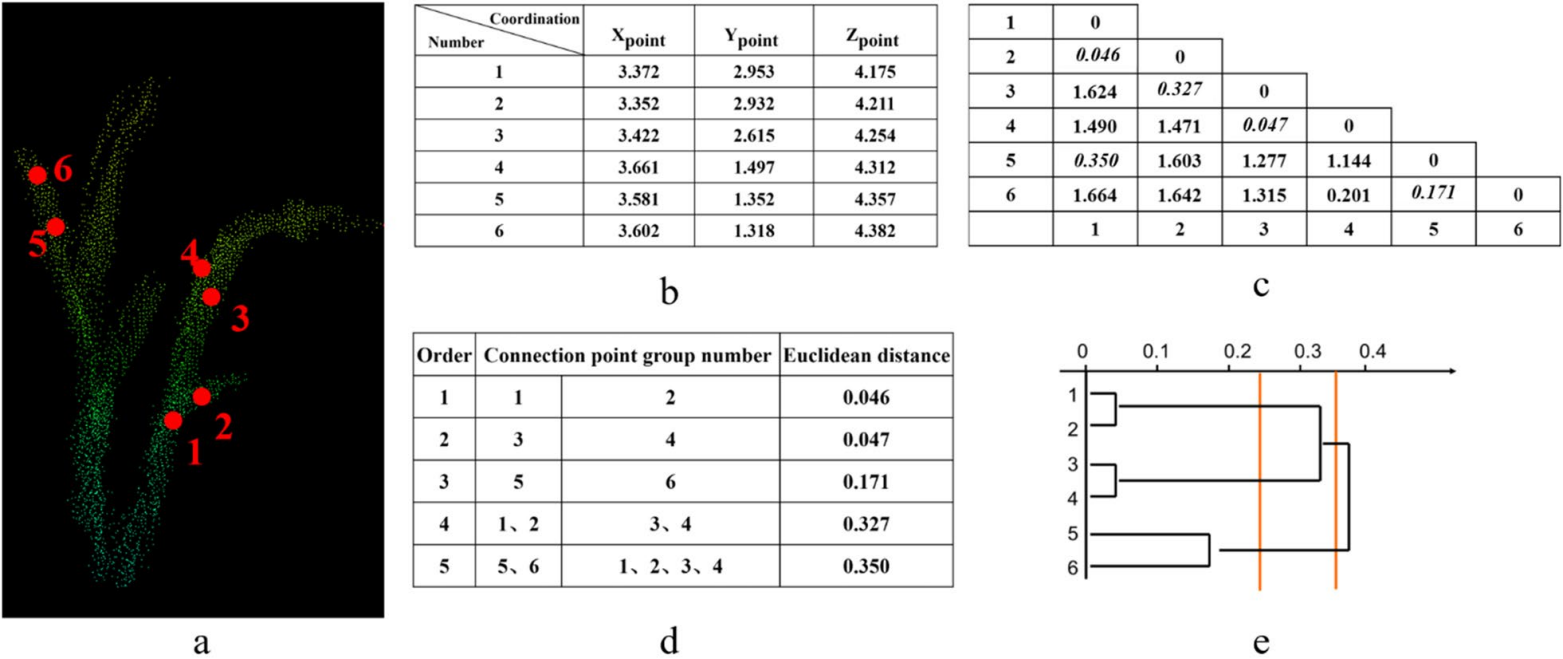
Point clouds clustered based on hierarchical clustering: **a** 3D point cloud of a cluster of wheat as an abstract sample, where the red dots marked with numbers represent sample points; **b** table of the original data; **c** the similarity matrix metric and the shortest Euclidean distance are marked with italicized numbers; **d** clustering order; and **e** the clustering diagram

(1) Calculate the similarity matrix of all points within a cluster. If one cluster contains N points, the similarity measurement matrix would be produced by N*N. Taking six random points (N = 6, and the matrix is 6*6) from the same cluster containing coordinate information (x_point_, y_point_, z_point_) as example to comprise a group of raw data (Fig. 6b). We obtain the similarity measurement matrix by calculating the similarity between each variable in the group.

The similarity measurement determines the similarity between two classes. Commonly used similarity metrics include the distance coefficient, correlation coefficient, and the cosine of angle. The appearance of tillers is very similar, where the cosine of angle may not be suitable for. Moreover, it is hard to define correlation within the unstructured and unordered point data, which is also not our choice. However, there are differences in spatial position. Therefore, we choose the Euclidean distance, a frequently used distance coefficient, as the similarity metric in this study. The specific formula is as follows

1 $${d}_{ij}=\sqrt{\sum\nolimits_{k=1}^{n}{\left({x}_{ik}-{x}_{jk}\right)}^{2}}, i, j=\mathrm{1,2},3\dots ,m$$

where *d*_*ij*_ is the Euclidean distance between the variables *i* and *j*, *n* is the dimension of variables *i* and *j*, and *x*_*ik*_ and *x*_*jk*_ are the data of the *k*th dimension for variables *x* and *j*, respectively.

(2) Calculate the Euclidean distance between all points, then merge the nearest two points into one class (Fig. 6c).

(3) Calculate the Euclidean distance between the newly generated class and other points, and end if the setting condition is met. Otherwise, continue the iteration until all points have been classified (Fig. 6d).

In our algorithm, the smallest unit is a cluster rather than a point. And the Euclidean distance *d* equaling 2 cm is set as the end condition of the iteration. We consider 2 cm as the minimum distance between two tillers based on the characteristics of wheat in field. To be discreet, we conduct a series of experiments to test the sensitivity of the algorithm to d. The total number of tillers per row is the sum of the number of tillers in each cluster, which is the cumulative value of all iteration result.

### Accuracy assessment

We evaluate the agreement between the tiller density computed with our method and the reference tiller density (field measurements) by the coefficient of determination (R^2^), calculated in Eq. (2). We also assess the precision of the computed tiller density with respect to the reference data using the root mean square error (RMSE) and the root mean square relative error (RRMSE), which are calculated with Eqs. (3) and (4), respectively, as follows:

2 $${\text{R}}^{2}\text{=1-}\frac{\sum_{\text{i} = \text{1}}^{\text{n}}{\left({\text{O}}_{\text{i}}-{\text{P}}_{\text{i}}\right)}^{2}}{{\sum }_{\text{i} = \text{1}}^{\text{n}}{\left({\text{O}}_{\text{i}}-\stackrel{\mathrm{-}}{\text{P}}\right)}^{2}}$$

3 $$\mathrm{RMSE}=\sqrt{\frac{1}{n}\sum_{i=1}^{n}{\left({O}_{i}-{P}_{i}\right)}^{2}}$$

4 $$\mathrm{RRMSE}=\frac{\sqrt{\frac{1}{n}\sum_{i=1}^{n}{\left({O}_{i}-{P}_{i}\right)}^{2}}}{\frac{1}{n}\sum_{i=1}^{n}\left({O}_{i}-{P}_{i}\right)}\times 100\%$$

where O_i_ is the i_th_ reference attribute measurement, P_i_ is the i_th_ computed attribute measurement and n is the total number of measurements being compared.

## Results

### Estimation of the wheat tiller with terrestrial LiDAR

The three experiments were used as examples of the estimation of the wheat tiller through terrestrial LiDAR with ALHC. Regarding the efficiency, the total calculation time was around 18 min on a PC with Intel Core i5-8265U CPU @ 1.6 GHz and 8 GB RAM, comparing with six hours one person of manual counting in our case. The time consumption of manual and TLS methods is summarized in Table 4.

**Table 4:** The time consumption of manual and TLS methods

Method		Time (36 plots)	Total time (36 plots)
Manual		6 h/person	6 h/person
TLS			
Scanning		1.6 h	2.6 h
Preprocessing		0.7 h	
Calculation		0.3 h	

**Fig. 7:**
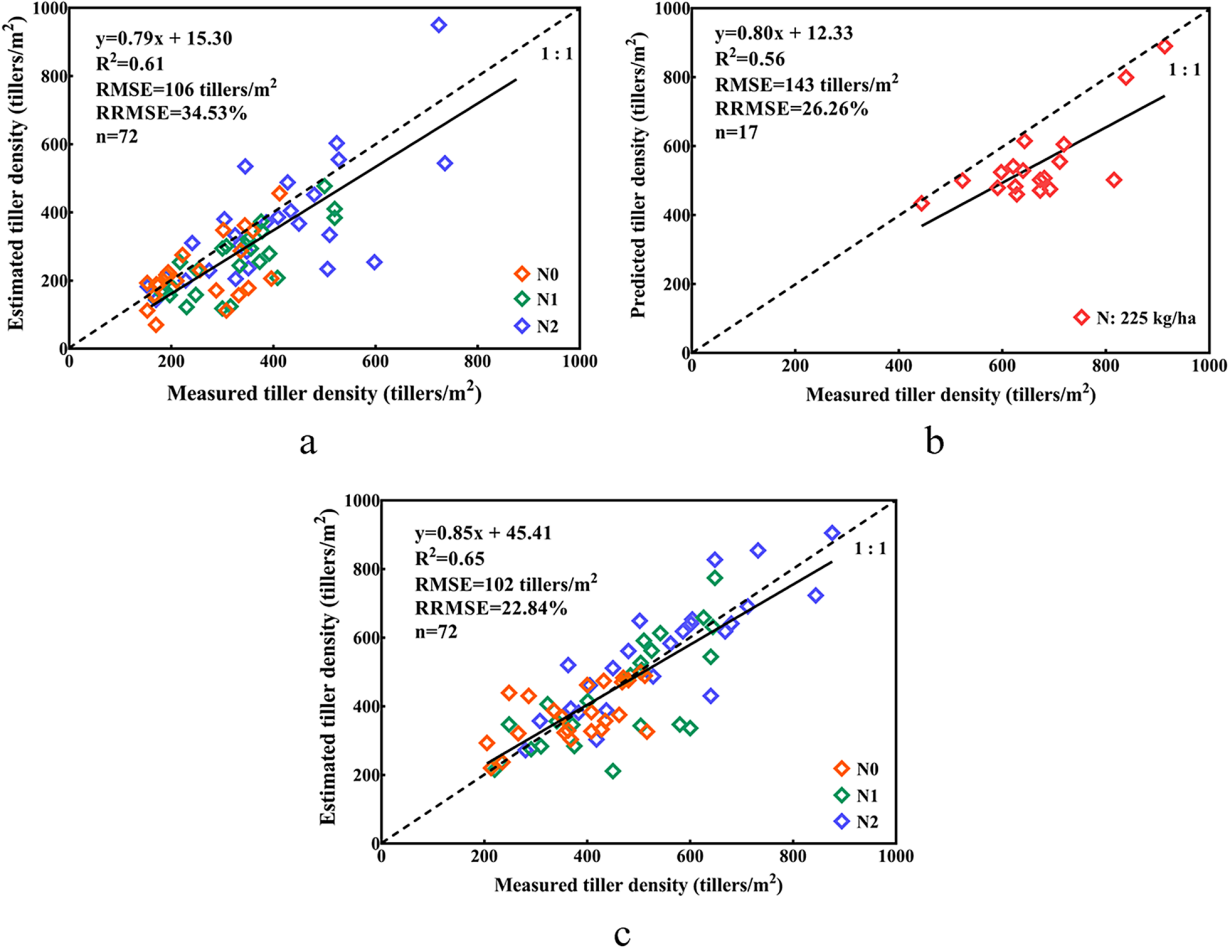
The tiller number estimation results for **a** Exp. A, **b** Exp. B, and **c** Exp. C; the dotted line is a 1:1 line; different colors represent different levels of nitrogen (Orange: N0, Green: N1, Purple: N2, Red: one nitrogen level between N1 and of N2 in Xuzhou)

The results showed performance (Fig. 7). Exp. C achieved the highest accuracy, with R^2^, RMSE, and RRMSE values of 0.65, 102 tillers/m^2^, and 22.84%, respectively. The lowest accuracy was observed in Exp. B (R^2^ = 0.56, RMSE = 143 tillers/m^2^ and RRMSE = 26.26%). The accuracy of Exp. A was of R^2^ = 0.61, RMSE = 106 tillers/m^2^ and RRMSE = 34.53%, which was at the same ecological site as Exp. C. Overall, there was a general underestimation of the ALHC estimation over all three experiments.

### Accuracy of the estimated tiller numbers in the various treatments

To evaluate the performance of the algorithm across multiple treatments, growth stages, and planting seasons, we formed a complete database with 2 years of data (Exp. A & C).

**Fig. 8:**
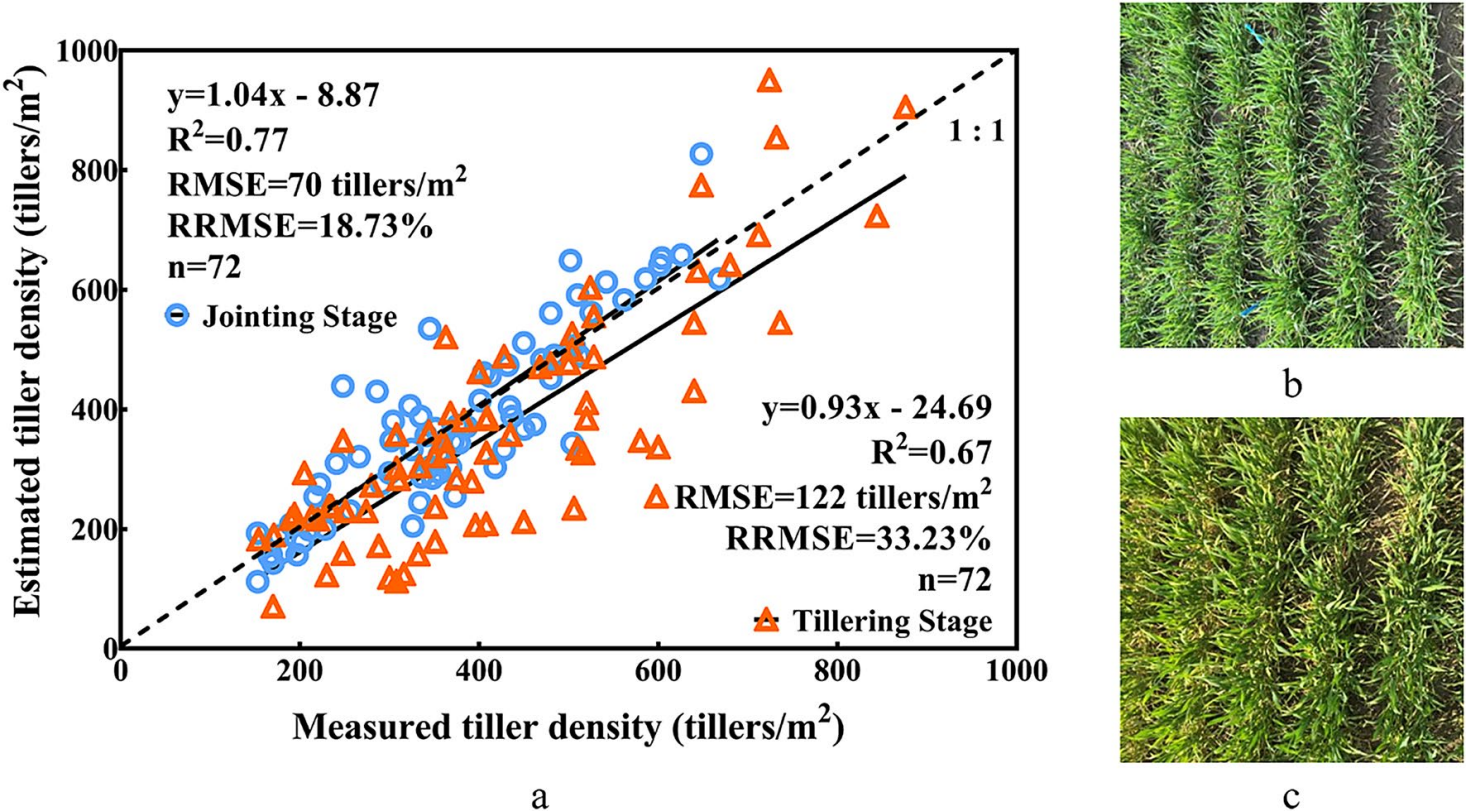
Comparison between the observed values and estimated values for the different growth stages (**a**). Field images of the plots under the same treatments (**b**: tillering stage; **c**: jointing stage)

First, to study the influence of the growth stages on the algorithm, we evaluated two years of data from the same ecological site (Rugao) for detailed comparison (Fig. 8). The results demonstrated that the correlation between the reference data and the jointing stage data (R^2^ = 0.77, RMSE = 70 tillers/m^2^, and RRMSE = 18.73%) was stronger than that with the tillering stage (R^2^ = 0.67, RMSE = 122 tillers/m^2^, and RRMSE = 33.23%). The growth stages had an important effect on the estimation accuracy, which revealed that the ALHC algorithm was more suitable for the jointing stage than the tillering stage.

**Fig. 9:**
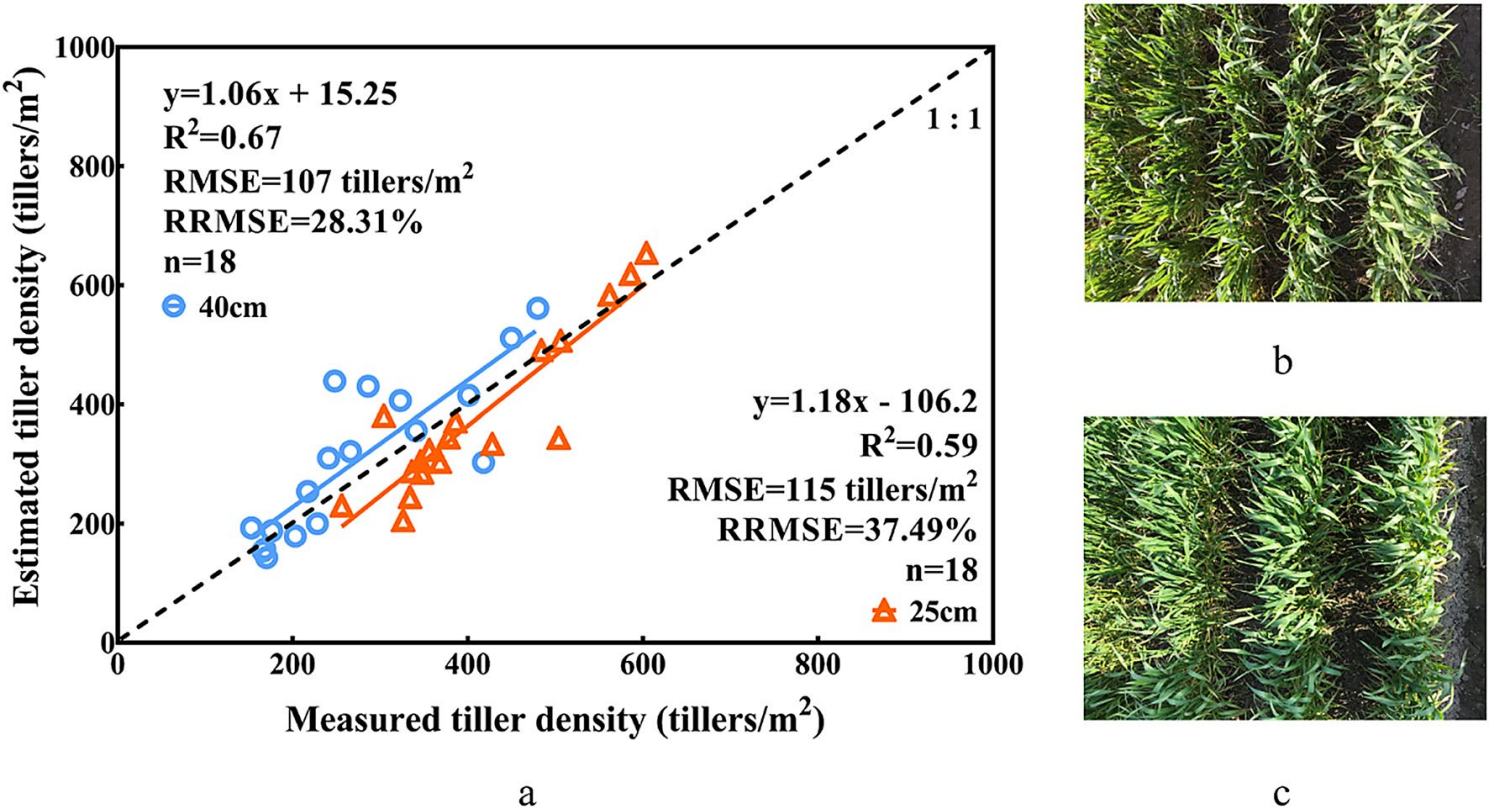
Comparison between the observed values and estimated values for the different planting densities (**a**). Field images of the plots under the same treatments (**b**: 25 cm; **c**: 40 cm) in the jointing stage

Second, to explore the influences of the planting densities (Fig. 9), two years of data of shared V2 with the two planting densities in the jointing stage were employed. As expected, higher accuracy was seen with lower planting density (R^2^ = 0.67, RMSE = 107 tillers/m^2^, and RRMSE = 28.13%). As the planting density increased, the accuracy was reduced (R^2^ = 0.59, RMSE = 115 tillers/m^2^ and RRMSE = 37.49%). The results indicated that the ALHC algorithm was negatively affected by planting density.

**Fig. 10:**
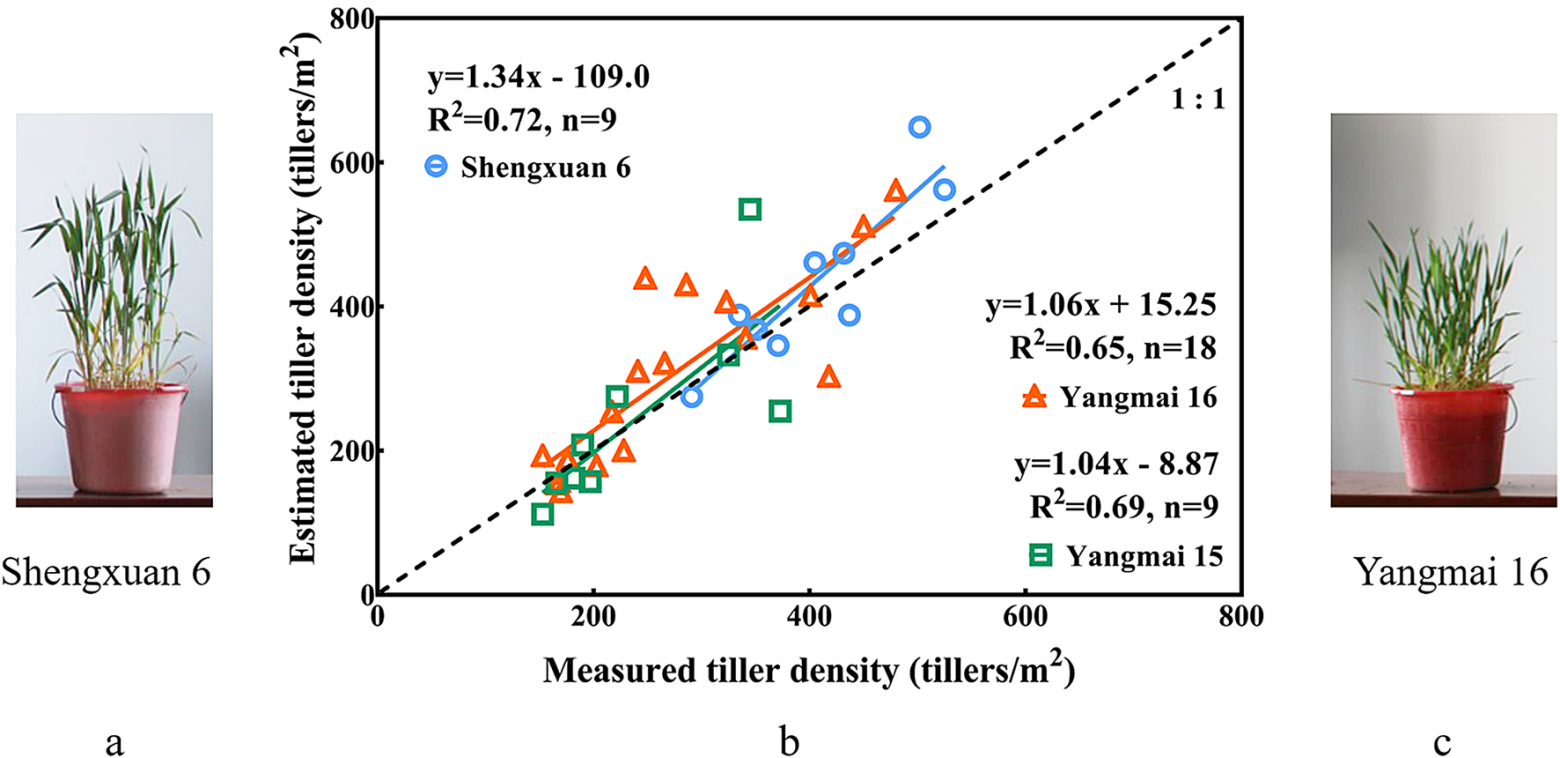
Comparison between the observed values and estimated values of the different varieties (**b**, Shengxuan 6: RMSE: 114 tiller/m^2^, RRMSE: 26.23%; Yangmai 15: RMSE: 167 tiller/m^2^, RRMSE: 38.43%; Yangmai 16: RMSE: 218 tiller/m^2^, RRMSE: 38.75%). Indoor images of the plants of different varieties (**a**: compact plant type: Shengxuan 6; **c**: loose plant type: Yangmai 16)

Finally, to explore the influences of the varieties, we employed two years of data of the jointing stage for the loose (Yangmai 16) and compact types (Shengxuan 6, Yangmai 15) (Fig. 10). Differences could be observed between the three varieties, which indicated that the loose plant type negatively impacted the estimation accuracy. Although Yangmai 15 and Shengxuan 6 were of the same plant type, the latter was estimated more accurately than the former.

Overall, although the different treatments affected the estimation accuracy of the ALHC, the accuracy fluctuated within a certain range.

In addition, we performed a series of experiments to explore the influence of the parameter (the Euclidean distance threshold d) in the algorithm on the results. Tanking the data from Exp. A (V2: Yangmai 16, D2: 40 cm, the jointing stage) as an example, the results were shown in Table 4. According to the numbers, the algorithm was sensitive to the changes of parameter. When *d* changed from small to large, the accuracy decreases first increased and then decreased. The trend implied that there was an optimal value to be explored (Table 5).

**Table 5:** The accuracy assessments of the ALHC on the example data from Exp. A (V2: Yangmai 16, D2: 40 cm, the jointing stage) with different d

d (cm)	R^2^	RMSE (tillers/m^2^)	RRMSE
0.5	0.48	170	46.38%
1.5	0.54	151	41.23%
2	0.60	136	37.11%
2.5	0.43	190	51.78%
3	0.30	272	74.21%

## Discussion

### Pros and cons of the ALHC method with TLS data

We automatically counted the tiller number based on TLS data under field conditions for the first time. Although improvements in the accuracy are required, the ALHC could provide a new perspective for subsequent studies. Compared with a previous study that used a single clustering algorithm [29], the ALHC considered the spatial structure of the wheat plants in the adaptive layering step, where the dense canopy was segmented into small groups according to the distribution of the spaces between plants. Another consequence of this step was to sort the disordered point clouds along the X axis, which made the subsequent clustering step more efficient. Although the classical k-means algorithm adopted in Guo’s study [29] was versatile, the hierarchical clustering algorithm had the advantage of fewer input parameters and the lack of a need to define the number of classes in advance [35, 36], which was more suitable for tiller counting. Combining these two steps, we upgraded from a single plant scale to a plot scale and from indoor laboratories to outside fields. In fact, the theoretical max measurement range of the TLS can be expanded by adding scanning sites. The stitching of point clouds from different sites would also achieve the broad coverage. For vast fields prepared for breeding selection, TLS is very practical. Moreover, the ALHC is expected to have the potential to be transferred to other cereal crops (e.g., rice) with similar spatial structures in the field.

The primary limitation of the algorithm is the method of determining the threshold. First, in the first part of the adaptive layering step, the height of every layer was chosen based on the degree of overcounting and undercounting of clusters that were observed in the layers compared with the planting density after the trail runs, which was an inefficient and biased method. Then, in the clustering step, the Euclidean distance threshold of ending the iteration in the clustering step was settled at 2 cm, which was considered to be the minimum distance between the clusters of wheat after field observation. However, the distance between the individual wheat plants will change as the crop grows, especially under different treatments. We could conclude from the trends (Table 4) that there were optimal values for the distances, which could be determined by the appropriate methods. A one-size-fits-all and subjective distance threshold may cause underestimation in high-density plots or overestimation in low-density plots, resulting in a decrease in overall accuracy. Future work will be devoted to developing objective methods for determining dynamically changing thresholds to eliminate empirical bias, such as the Otsu thresholding method [37] and the probability density function (pdf). Moreover, a few variants and alternatives of the hierarchical clustering algorithm have been suggested for improving the function of the algorithm [33, 38], which may help with the algorithm performance under large numbers of disordered points and the accompanying noise.

### Uncertainty of the ALHC counts of tiller number based on TLS data

This method has not only remedied the deficiency of passive remote sensing methods through using spatial information of the 3D point clouds but also filled the gap in counting the tiller number in the field. However, there were still some shortcomings. Compared with the previous studies [8–13, 15, 16, 29], the accuracy of the TLS data was unexpectedly low. The main reasons could be concluded from two aspects: (1) noise and (2) occlusion, which are currently inevitable in the TLS data.

It has been proven that noise negatively impacts TLS-derived parameters [39]. Noise may be generated by the common registration differences of point clouds, multiple echoes for one emitted shot, or instrumental errors associated with the measurement range [40]. Because it is more complicated than a single background laboratory, the field environment was surrounded by multisource noise, such as wind-caused noise. In our case, the denser the canopy was (the higher the planting density was), the more noise points it contained, which was intuitively reflected in the reduced accuracy. In contrast, more noise came from the edges of the crop foliage and the ground in the tillering stage, where it was covered by leaves in the jointing stage, which would contribute to higher accuracy [26]. In addition, noise was also one of the main reasons for overestimation. And the other was due to the incorrect recognition of leaves as tillers in the clustering step. Robust noise filtering algorithms are needed to make TLS a more powerful tool for crop field phenotyping.

Occlusion causes a significant loss of data. When the laser beam was intercepted by canopy elements, the space behind these elements was not sampled, which is called occlusion. In contrast to the individual plants monitored in the indoor laboratory, the overlap between and inside the plants was extremely challenging in the outdoor field, which prevents the laser from penetrating the canopy to obtain information from the middle and lower parts of the plant. In our case, for example, the accuracy declined with the changes in wheat varieties from a compact type to a diffuse type, where the occlusion was more serious in the latter. The same decrease in accuracy could also be observed with increased planting density. The incomplete canopy information caused by occlusion could also explain the general underestimation among our experiments (Fig. 7). Since the TLS was mounted on a tripod with a height of 2 m, the laser pulses stroke the plants at large incident angles (from 40° to 90°). To improve the laser’s penetration of the dense canopy, it would be beneficial to use a close to a nadir viewpoint instead of a gentle oblique viewpoint [41]. The airborne LiDAR could provide a smaller incident angle, which could penetrate the canopy more powerfully. Compensating for the loss of point cloud information by increasing the instrument height and increasing the number of scanning sites could also alleviate the problem [38].

In addition to the above methods, it is worth paying attention to methods based on machine learning, which could be tailored to learn complex features from large amounts of high-dimension data automatically [42]. These have the potential to outperform traditional methods.

## Conclusion

This study proposed an automatic and nondestructive method for automatically counting wheat tillers in the field with terrestrial LiDAR data, which first separated the clusters with the adaptive layering algorithm and then detected the tillers in every cluster with the hierarchical clustering algorithm. The results showed that the ALHC method was promising and had the adaptability across different years, growth stages, planting densities, and ecological sites. However, due to the serious occlusion between and inside the plants and the numerous noise points, there was a general underestimation of the wheat tillers. The counting accuracy still needs to be improved in the ongoing work of replacing the missing points in the cloud and eliminating the noise points in the cloud. It would be meaningful to transfer the improved ALHC method to other cereal crops (e.g., rice) to potentially enhance the universality of the tiller counting algorithm.

## Data Availability

The datasets used and/or analyzed during the current study available from the corresponding author on reasonable request.
